# Optimizing Feeding Regimes and Vitamin Delivery Methods in Microdiet for Improving Survival and Growth of Carp Larvae

**DOI:** 10.1155/anu/3026254

**Published:** 2026-06-29

**Authors:** Zsuzsanna J. Sándor, László Ardó, Jelena Stanivuk, Attila Terhes, Shivendra Kumar

**Affiliations:** ^1^ Research Centre for Fisheries and Aquaculture, Hungarian University of Agriculture and Life Sciences, Szarvas, Hungary, uni-mate.hu; ^2^ Department of Aquaculture, College of Fisheries, Dr. Rajendra Prasad Central Agricultural University, Dholi, Bihar, India

**Keywords:** digestible proteins, encapsulation, plant-based diet, sparing effect, stress resistance, vitamins

## Abstract

In pond carp production, there is growing interest in producing larvae out of season to shorten the production cycle and increase profitability. For this reason, carp fries are produced in indoor recirculation systems, where larvae are fed *Artemia salina*. Due to the limited ability of fish larvae to digest conventional microdiets—attributable to their short, agastric intestines—live feed remains essential. Therefore, the encapsulation of micronutrients, such as vitamins, may improve delivery efficiency. To address these challenges, three trials were conducted with the following aims: (i) to develop the most effective weaning strategy; (ii) to evaluate the best encapsulation method for vitamin delivery to larvae; (iii) to test different protein sources in microdiets; and (iv) to assess the effectiveness of vitamin supplementation under stress conditions. The data on production parameters demonstrated that feeding common carp larvae with *Artemia* for 8 days, followed by a 4‐day co‐feeding period, is necessary to initiate the utilization and nutrient uptake from microdiets. Regarding vitamin delivery via encapsulation technology, better performance was achieved when proteins were used as encapsulating agents compared to oil‐based carriers. Additionally, the survival rate of fish fed with plant‐based microdiets was significantly lower (66.2% ± 11.2%) than that of larvae fed with fish‐based diets (91.1% ± 3.0%) at 32 days post‐hatch (dph). With respect to vitamin supplementation, enrichment with water‐soluble vitamins (WSVs) was not detected in fish larvae; however, a slight association was observed between antioxidant vitamin levels and stress markers. In conclusion, fish‐based microdiets show strong potential as a substitute for live feed in indoor carp larval rearing from 12 dph onward. However, further development of the encapsulation process is necessary to improve the delivery efficiency of WSV.

## 1. Introduction

In the last century during decades, a great deal of interest has been generated in the development of a microdiet as an economic live food alternative, initially for marine fish larvae [1–4]. It was aimed that replacing live prey production and fertilized ponds for hatchery with a storage room of high‐quality micro particulate diets would increase seed production, improve the consistency of hatchery production, and lower the costs of production in many aquaculture species. Similarly, production of live prey in bioreactors has several weaknesses emphasizing the importance of turning artificial dry feeds in reliable larviculture production. Despite these needs and expectations, still today larval rearing of the intensive cultured freshwater and marine fish species depends on live food (algae, rotifers, and *Artemia salina*) [5–7]. As a significant factor hindering the success of solely using artificial diets, the limited digestive capacity of fish larvae at the onset of exogenous feeding has been identified [8–10]. Although microdiets developed exclusively for feeding marine fish larvae performed poorly, their results improved significantly when inert diets were supplemented with live *Artemia* nauplii [11, 12]. By the way, it was observed that microdiet supplemented with various fractions extracted from *Artemi*a nauplii, that is, neutral and polar lipid classes or a non‐lipid fraction, separately and in combination, significantly increased microdiet assimilation by 10%–20%. Similarly, 30% increase in digestibility could be achieved by exogenous enzyme supplementation of the microdiets [11] or adding free amino acids (glycine, alanine, and arginine) by stimulating the larvae appetite. In stomachless cyprinid species, digestion occurs in the intestine through digestive enzymes, the levels of which depend not only on the larvae’s ontogeny but also on the composition of the diet provided [13, 14]. Therefore, initiating feeding should be based on an understanding of the development of the digestive system [15], as larvae must be physiologically capable of digesting the consumed food [16].

Common carp is the most important fish species in Hungarian fish farming. For most freshwater aquaculture species, one of the main bottlenecks is the stable production of high‐quality juveniles. In traditional extensive carp production, high and unpredictable mortality rates (sometimes exceeding 80%) occur in the first weeks after hatching. Recently, there has been increased interest in combined fish farming technology, where larvae are produced off‐season and reared in intensive closed recirculating systems [17, 18]. High interest in producing larvae out of season contributed to shortening the production cycle and increasing the profitability of aquaculture. In the in‐door larviculture of common carp, it still remained a challenge to replace live prey with formulated microdiet to reduce costs and increase predictability of juvenile production.

The progress in designing microdiets today is linked to advances in both the microparticulation technology and the knowledge on nutrition, feeding physiology, and feeding behavior of larval fish. From a practical perspective, the microdiet preparation method to be used in the future should be able to include in the food particle a complete dietary formula and use practical ingredients. Different protein hydrolysates are considered promising protein suppliers for larvae beside being feed attractants and also as palatability enhancers for fish [19, 20]. Therefore, the ratio between water soluble and insoluble protein in the diet needs to be balanced according to the developmental stage of the larvae [9]. Microencapsulated diets might be the future starter food for common carp, offering a more stable quality compared to live food. In microencapsulation, covering the nutrient mixture with a capsule wall reduces nutrient leaching or oxidation [3, 21]. However, this methodology may reduce diet ingestion rates precisely by reducing the release of nutrients into water. Besides, the capsule wall must be highly digestible by larvae whose digestive systems are not completely developed. Microencapsulation can be achieved either by chemical (spraying on solid, gas, or liquid phases) or mechanical methods. Despite their relatively low efficiency of micronutrient inclusion, protein‐walled microencapsulated diets can retain sufficient compounds when immersed in water and deliver them to the larvae’s digestive tract [22].

According to earlier findings, the survival and growth of larvae alone might not be sufficient to optimize the feeding strategy based on microdiets [12, 23]. Assessing survival rates and the accumulation of micronutrients, such as vitamins, during periods of acute stress could provide additional interesting information. Therefore, this study was conducted to evaluate the effect of starter diets for weaning the common carp larvae using innovative delivery methods for water‐soluble vitamins (WSVs). For this reason, the objectives were set: (i) to develop the most effective weaning strategy; (ii) to evaluate the best encapsulation method for vitamin delivery to larvae; (iii) to test plant protein and fat sources in microdiets; and (iv) to assess the effectiveness of vitamin supplementation under stress conditions.

## 2. Materials and Methods

### 2.1. Ethics (Institutional Review Board Statement)

All animal research mentioned in this manuscript was carried out in accordance with European Union Council (2010/63/EU) criteria, which were approved by the institute’s Ethical Committee (1/2002) in accordance with Hungarian State law (10/1999. [I.27.] and 40/2013. [II.14.]).

### 2.2. Experimental Setup

The objectives of the present study were addressed through the following three experimental trials:

Experimental trial 1: Developing a weaning strategy to enhance the utilization of microdiets for carp larvae.
Experimental trial 2: Formulation of an effective microdiet to improve the growth and survival of carp larvae.
Experimental trial 3: Evaluating microdiets for their effectiveness in delivery WSVs to enhance the stress resistance of carp larvae.

#### 2.2.1. Experimental Trial 1: Developing Weaning Strategy for Better Utilization of Microdiet by Common Carp Larvae

##### 2.2.1.1. Experimental Diet

The microdiet for the first feeding of carp larvae was prepared at SPAROS in Faro, Portugal. The diet was formulated using marine protein hydrolysates according to [19, 23, 24] and was supplemented with 100 mg kg^−1^ ascorbate phosphate, 12,000 IU kg^−1^ vitamin A, and 250 mg kg^−1^ vitamin E. These vitamins have been previously encapsulated to avoid leaching according to Link and Schündler [25] by using Nero Aeromatic (Bubendorf, Switzerland). The main ingredients were grinded (below 200 micron) in a micropulverizer hammer mill (Hosokawa Micron, SH1, The Netherlands) thoroughly mixed with fish oil and marine phospholipids. The diet was manufactured by temperature‐controlled extrusion by means of a low‐shear extruder (Italplast P55, Italy) and dried in an oven at 40°C for 24 h. The pellets were then ground in a centrifugal mill (Retch ZM 200, Germany) and manually sieved to obtain the desired particle size. Composition of the diet on original matter is as follows: crude protein (CP) 56%, crude fat 12%, crude fiber 0.9%, crude ash 12.8%, and gross energy 21.3 MJ kg^−1^. The dry microdiets were sieved through adequate nets to obtain two fractions: from 200 to 400 µm and from 400 to 600 µm. These diameter ranges fit well to the preferred particle size of carp larval fish during the first weeks of feeding.

##### 2.2.1.2. Experimental Design

**Figure 1:**
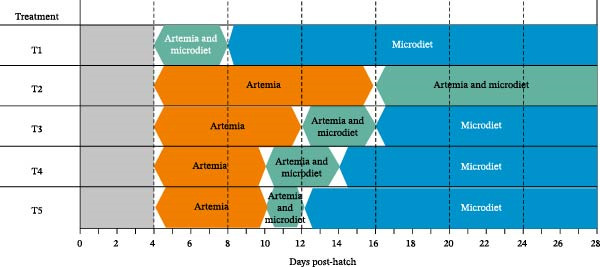
Feeding schedule.

The 24‐day experiment was conducted with 3 days old common carp larvae (initial size 1.89 ± 0.04 mg) in January 2015. The larvae were reared in 45 L tanks in the RAS system of the institute, where the water temperature was between 21–23°C. The stocking density was 1000 fish tank^−1^. The trial was set up based on different feeding strategies using as live feed *Artemia* nauplii and as co‐feeding diet the microdiet presented. Such feeding protocol was designed so that the co‐feeding period with *Artemia* nauplii was decreased (Table 1 and Figure 1). Five treatments were distinguished and distributed in triplicate. The feeding was carried out by hands, following the feeding protocol four times per day to visual satiation. In case of co‐feeding, the fish were fed each day two times with *Artemia* and two times with microdiet. The amount of live feed was set to be freshly hatched nauplii with a density of 4–5 ind mL^−1^. Cleaning of the tanks was performed three times per day using a salt‐soaked brush. The waterflow was set to change the water in tanks every hour. The water quality (dissolved oxygen, pH, conductivity, and temperature) parameters were recorded daily by a portable multimeter, while physicochemical properties (total ammonia nitrogen, unionized ammonia nitrogen, and nitrite nitrogen) were measured in accredited laboratory two times per week. The dissolved oxygen level was kept above 80% saturation, conductivity increased from 1200 to 6210 μS cm^−1^, ammonia‐N was below 0.1 mg L^−1^, and similarly, nitrite‐N was below 0.035 mg L^−1^. Nitrate‐N varied between 6.12–8.65 mg L^−1^, while pH varied between 8.07 and 8.96, respectively.

**Table 1:** Feeding regime of larvae after starting the exogenous feeding (4 day post‐hatch‐dph).

Treatments	Artemia	Co‐feeding	Microdiet
	Days of feeding		
Treatment 1 (T1)	0	4	20
Treatment 2 (T2)	12	12	0
Treatment 3 (T3)	8	4	12
Treatment 4 (T4)	6	4	14
Treatment 5 (T5)	6	2	16

##### 2.2.1.3. Stress Experiment

The stress experiment was implemented using all groups, except for T1. Larvae from the first T1 group did not take part in the stress experiment because of the low growth and survival rate parameters. The larvae were fasted for 12 h before stress implementation. First, confinement circumstances were established by reducing the water depth from 22 to 2 cm for 1 h. During this time, the temperature decreased from 22 to 13.5°C due to the suspension of the heating. The digestible oxygen level at 1 h after the stress situation varied between 72%–84%, the lowest level detected in the tanks containing the T2 group. After the stress experiment, the larvae were kept further for 4 days in order to check the survival rate and the antioxidant vitamin levels of fish following a recovery session (Figure 1). The larvae were kept on the same rearing conditions as before and fed according to the protocol described (four times per day to visual satiation).

##### 2.2.1.4. Sample Collection

The sampling was performed on larvae that had been fasted for 12 h, typically at 8:00 a.m., considering that the last feeding had occurred at 8:00 p.m. on the previous day. During the nutritional trial, weight and length were recorded individually (initial *n* = 50 tank^−1^, intermediate and final sampling *n* = 25 ind tank^−1^), and at the end of trial, samples have been taken for determination of body composition. For the analytical measurements, 25–125 individual fish per treatment (10 g) were collected as pooled samples, promptly frozen, and then stored at −80°C until analysis.

#### 2.2.2. Experimental Trial 2: Formulation an Effective Microdiet to Improve the Growth and Survival of Carp Larvae

##### 2.2.2.1. Experimental Diet

In order to formulate the most digestible micro diet for carp larvae, six iso‐nitrogenous and iso‐lipidic diets with different ingredient sources (diets with marine origin FB‐CTRL, FB‐P, and FB‐O and plant origin PB‐CTRL, PB‐Pr, and PB‐O) and encapsulation material (none capsulated diets, FB‐CTRL and PB‐CTRL; protein encapsulated diets, FB‐Pr and PB‐Pr; and oil encapsulated diets, FB‐O and PB‐O) were formulated and produced (SPAROS, Faro, Portugal). The diets were formulated to contain 60% protein with 45% water‐insoluble protein + 15% water‐soluble protein according to Carvalho et al. [9]. The gross energy content was planned to reach 20 MJ kg^−1^ and the ash level below 10%. The encapsulation was performed by spray drying, when a blend of several WSVs (thiamin, riboflavin, niacin, pyridoxine, folate, biotin pantothenic acid, vitamin B12, vitamin C, and vitamin E) on similar concentration level in two distinct matrices were administered. These matrices were protein‐based (fish gelatin/whey protein isolate) and lipid‐based (oil/lecithin). Formulation of the basic diets using different protein sources is presented in Table 2. Proximate compositions of all diets determined are presented in Table 3.

**Table 2:** Formulation of the basic feeds using two groups of ingredient sources (FB and PB).

Ingredients (%)	FB	PB
Micronised FM LT 70	46.00	—
CPSP 90	13.00	—
Squid meal	13.00	—
Soy protein isolate	—	23.00
Pea protein concentrate	—	17.50
Wheat gluten	—	17.50
Peas gelatinized (Aquatex 8071)	5.801	3.531
Fish oil	2.00	—
Krill oil	7.65	—
Linseed oil	—	6.30
Soy lecithin	—	8.20
Mineral premix HAKI	1.00	1.00
NaH2PO4	2.20	5.50
Rovimix A500	0.008	0.008
Rovimix D3	0.0003	0.0004
Vitamin K	0.0002	0.0002
Lutavit E50	0.040	0.040
Choline chloride	0.600	0.600
Vitamin premix HAKI	1.000	1.000
L‐arginine	—	0.800
L‐histidine	—	0.700
L‐isoleucine	—	0.700
L‐leucine	—	0.350
L‐Lysine	—	2.100
L‐threonine	—	0.750
L‐tryptophan	—	0.520
L‐valine	—	0.600
DL‐methionine	—	0.700
Taurine	—	0.900
Binders, antioxidants, humectant	7.700	7.700
Total	100.0	100.0

*Note*: Vitamin premix HAKI: 0.5 mg kg^−1^ thiamin, 6 mg kg^−1^ riboflavin, 6 mg kg^−1^ B_6_, 40 mg kg^−1^ pantothenic acid, 28 mg kg^−1^ niacin, 1 mg kg^−1^ biotin, 0.05 mg kg^−1^ vitamin B_12_, 1 mg kg^−1^ folate, and 50 mg kg^−1^ vitamin C.

**Table 3:** Proximate compositions of the experimental diets used in trial 2 (as is).

Treatments	FB‐CTRL	PB‐CTRL	FB‐Pr	PB‐Pr	FB‐O	PB‐O
Water (%)	1.48	2.06	4.42	4.19	5.25	5.66
Crude protein (%)	63.18	63.90	62.66	60.98	62.34	62.38
Crude fat (%)	12.79	13.11	11.82	11.87	12.17	11.60
Crude ash (%)	11.09	13.98	11.10	11.20	13.35	10.71
Crude fiber (%)	0.63	0.23	0.41	0.47	0.39	0.33
Nitrogen free extract (%)	10.83	6.72	9.59	11.28	6.50	9.32
Gross energy (MJ kg^−1^)	21.79	21.37	21.08	21.00	20.60	20.88

*Note*: FB‐CTRL, fish‐based diet none caps; PB‐CTRL, plant‐based diet none caps; FB‐Pr, fish‐based diet with protein caps; FB‐O, fish‐based diet with oil caps; PB‐Pr, plant‐based diet with protein caps; PB‐O, plant‐based diet with oil caps.

##### 2.2.2.2. Experimental Design

Experiment was carried out in July 2015, when 3‐day‐old carp larvae were placed in a 50 L‐aquarium system at HAKI in three replicates for six different diets (Table 2). The stocking density was 100 fish aquarium^−1^, and the water temperature ranged between 22–24°C. The carp larvae were fed with *Artemia* nauplii for 8 days (12 days post‐hatch [dph]), and co‐feeding with artificial diet lasted for 4 more days (feeding regime T3). From 16 dph, the larvae were fed only with the experimental diets. Feeds were manually supplied each 60 min from 5:00 to 22:00 and every 2 h during the night till satiation. All aquariums were thoroughly cleaned with a salt‐soaked brush and partly water exchange administered every day. Water quality was monitored as described in the first trial. The following average values were recorded: conductivity 608 μS cm^−1^, ammonia‐N 0.131 mg L^−1^, nitrite‐N 2.03 mg L^−1^, nitrate‐N 0.652 mg L^−1^, and pH 7.99. Temperature ranged between 25 and 26.2°C, and dissolved oxygen levels were maintained above 85% saturation. Final weight (mg), feed utilization, and survival (%) were assessed after 26 days of hatching (after 10 days of feeding trials). The sample collection was performed as previously described in Section 2.2.1.4.

#### 2.2.3. Experimental Trial 3: Effectiveness of Weaning Diet for Delivery of WSVs

##### 2.2.3.1. Experimental Diet

The microdiets for feeding of common carp larvae were produced by SPAROS based on the results obtained from previous experiments presented in experimental trial 2. For the microencapsulation of vitamin E,different levels of WSVs (1x, 2x, and 3x of the vitamin requirement of carp larvae) were used in the protein matrix. The ingredient sources were of plant and marine origin, using the formulation presented in previous trial (FB and PB) (Table 2) and depicted as FBD1x–FBD3x diets and PBD1x–PBD3x diets in this trial. The proximate composition of six micro diets as well as the supplemented vitamin concentration of diets is presented in Table 4. The fatty acid composition of the diets is summarized in Table 5.

**Table 4:** Proximate compositions and vitamin content of the experimental diets used in trial 3 (as is).

Treatments	FBD1x	FBD2x	FBD3x	PBD1x	PBD2x	PBD3x
Proximate composition						
Water (%)	7.24	7.53	7.39	7.72	8.56	7.80
Crude protein (%)	66.12	66.34	66.82	63.69	64.74	65.46
Crude fat (%)	13.00	12.79	12.92	13.69	12.52	12.01
Crude ash (%)	11.21	11.03	11.24	10.80	11.02	9.36
Crude fiber (%)	0.46	0.69	0.71	0.24	0.32	0.41
Nitrogen free extract (%)	9.21	9.15	8.32	11.58	11.40	12.75
Gross energy (MJ kg^−1^)	21.02	20.93	20.97	21.04	20.66	21.00
Vitamin supplementation level (mg kg^−1^)						
Vitamin C	50	100	200	50	100	200
Vitamin B complex	1x	2x	3x	1x	2x	3x
Vitamin E	100	100	100	100	100	100

*Note*: FBD1x, FBD2x, and FBD3x, fish‐based diets. PBD1x, PBD2x, and PBD3x, plant‐based diets. Vitamin B complex: 0.5 mg kg^−1^ thiamin, 6 mg kg^−1^ riboflavin, 6 mg kg^−1^ B_6_, 40 mg kg^−1^ pantothenic acid, 28 mg kg^−1^ niacin, 1 mg kg^−1^ biotin, 0.05 mg kg^−1^ vitamin B_12_, and 1 mg kg^−1^ folate.

**Table 5:** Fatty acid profile of the diets (as is).

Treatments	FBD1x	FBD2x	FBD3x	PBD1x	PBD2x	PBD3x
Fatty acid composition (w%)						
14:0	2.72	2.67	2.67	0.14	0.13	0.12
16:0	18.22	18.29	18.24	14.43	14.55	14.43
16:1n‐7	3.84	3.70	3.75	0.17	0.14	0.13
18:0	3.04	3.10	3.11	4.00	4.03	3.91
18:1n‐9	14.66	14.69	14.67	18.26	18.37	18.12
18:1n‐7	2.48	2.45	2.48	1.25	1.23	1.25
18:2n‐6	16.23	15.85	15.72	37.28	37.45	37.89
18:3n‐3	2.34	2.30	2.29	21.99	21.54	21.74
20:4n‐6	0.67	0.66	0.66	0	0	0
20:5n‐3	7.07	6.90	6.96	0.11	0.11	0.10
22.6n‐3	13.11	13.01	13.20	0	0	0
Total SAT	24.88	25.02	24.97	19.32	19.49	19.24
Total MUFA	26.48	26.75	26.73	20.00	20.09	19.84
Total PUFA	41.03	40.41	40.47	59.71	59.24	59.89

*Note*: FBD1x, FBD2x, and FBD3x, fish‐based diets. PBD1x, PBD2x, and PBD3x plant‐based diets. SAT‐saturated fatty acids, MUFA‐monounsaturated fatty acids, PUFA ‐polyunsaturated fatty acids.

Abbreviations: MUFA, monounsaturated fatty acids; PUFA, polyunsaturated fatty acids; SAT, saturated fatty acids.

##### 2.2.3.2. Experimental Design

The experiment was conducted similarly in the RAS system of HAKI between May–June 2016. The feeding trial started at 12 dph with co‐feeding with *Artemia* for 4 days and totally lasted for 20 days (32 dph), following the results of previous experiments. The stocking density was 1000 fish tank^−1^, and six experimental feeds were applied in three replicates. The larvae were reared in 45 L tanks, and the water temperature was kept between 21–22°C. Initial average weight of common carp larvae (12 dph) was 12.68 ± 0.72 mg. Fish were fed ad libitum by hand hourly during daylight from 5 to 10 pm and every 2 h at night. The dissolved oxygen level was kept above 80% saturation. Conductivity ranged between 1540 and 1715 μS cm^−1^, ammonia‐N remained below 0.1 mg L^−1^, nitrite‐N below 0.02 mg L^−1^, nitrate‐N ranged between 16.8 and 19.3 mg L^−1^, and pH was 8.52. At the end of the trial, larvae were sampled for growth parameters (30 ind tank^−1^) and vitamin retention of the larvae (50 ind tank^−1^). They were collected after 12 h of fasting prior to the sampling.

**Figure 2:**
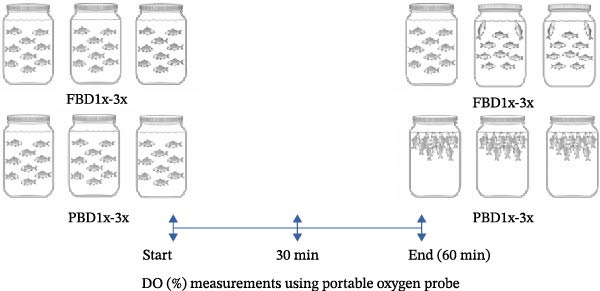
Schematic design of the hypoxia condition.

To study the stress defense system of the fish, the following two different stress challenge tests were conducted:

Confinement stress: Modeling of the transport conditions fish were exposed to confinement stress to study the effects of vitamin consumption. At the end of the feeding trial (on the 32 dph), after 12 h of fasting the larvae, water columns were decreased from 20 to 2 cm, and fish were kept in this condition for 2 h. The initial water temperature was between 21.7–22°C and remained relatively constant (21.8–22.7°C) at the final sampling. The concentration of oxygen was decreasing during the stress from 90.3% ± 2.5% to 20.3% ± 12.8% dissolved O_2_. Whole fish were sampled carefully for vitamin analysis on ice before the stress event, immediately after the stress, and 4 days later and kept refrigerated at −80°C till analysis. Other batches of whole larvae samples were collected in RNA later for gene expression studies before and after stress administration.
Hypoxia stress: Hypoxic conditions were induced on the same day as the confinement stress challenge was done using the same aged fish. For this trial, 1 L jars were filled with tap water without any pre‐treatments, and 15 larvae tank^−1^ were placed into each jar (Figure 2). Dissolved oxygen levels at the beginning of the trial ranged between 0.43% and 0.67%, while water temperature was 24.3°C. Dissolved oxygen concentrations were measured after 30 and 60 min by carefully inserting the probe of a portable multimeter into each jar individually, confirming that oxygen levels remained between 0.71% and 0.92%. No additional precautions were taken to prevent oxygen diffusion from the air. During the hypoxia treatment, the number of air‐gulping larvae on the surface was recorded every 5 min, as common carps can survive severe hypoxic conditions by aquatic surface respiration (ASR) or taking oxygen directly from the air [26–28].

### 2.3. Analytical Methods

#### 2.3.1. Proximate Composition Analysis

The chemical composition of feeds (Tables 1–3) was analyzed by standard methods of the AOAC [29]. CP was determined by the Kjeldahl AOAC 928.08 method [29] using a digestion block (KJELDATHERM, Gerhardt, Germany) via a distillation procedure (VAPODEST 450, Gerhardt, Germany). Approximately 0.5 g of diet and 1 g pooled larvae samples were digested with 10 mL of 98% H_2_SO_4_ and 10 mL of 30% H_2_O_2_, and subsequently, the generated ammonium sulfate was distilled off using 2% H_3_BO_3_. The CP was calculated as *N* × 6.25 for diets and feces. The crude fat was determined from 5 g of dry diet samples according to the AOAC 945.16 Soxhlet method [29] using an automatic system (SOXTHERM Unit SOX416, Gerhardt, Germany) and diethyl ether (boiling point, 40–60°C) as a solvent. The crude ash content of the diets was estimated according to the AOAC [29] 942.05 method. 2 g of the samples was weighed and placed in a furnace heated to 550°C and held for 4 h. The amount of ash that remained was recorded. For diet crude fiber determination [29], 1.5–2.0 g were used, and the digestion procedure was carried out using 0.13 M H_2_SO_4_ and 0.31 M NaOH in a GERHARDT Fibretherm FT12 apparatus (Königswinter, Germany). The gross energy value of the experimental diets was determined using [30].

#### 2.3.2. Chromatographic Methods for Determination of Vitamins

Vitamin C, B_1_, and B_6_ concentrations of *Artemia* and common carp larvae were determined by an isocratic reverse phase HPLC method developed for vitamin C determination [31] and standardized for vitamin B_1_ and vitamin B_6_ (Gy. Papp unpublished results). Pooled larvae (4–5 individuals, ~min 1 g) were deproteinized with perchloric acid after extraction with sodium acetate buffer (pH 4.8) and used for the determination of L‐ascorbic acid. The whole L‐ascorbic acid content was oxidized with a specific enzyme reaction (ascorbate oxidase) to do a background correction. Dehydro‐L‐ascorbic acid was reduced with 1,4‐dithioerythritol to L‐ascorbic acid to determine the total vitamin C concentrations. The concentrations of B vitamins were calculated using the peaks of all fractions. Chromatograms were developed on a Waters HPLC system using 0.04 mol L^−1^ sodium acetate, with 0.05 mmol L^−1^ EDTA‐t and 0.5 mmol L^−1^ TBA buffer at pH 3.76; Nova‐Pak C‐18 column 30 cm × 3.9 mm i.d. at 23 °C, and detection was carried out with a UV DAD detector at 250 nm.

The vitamin E (alpha‐tocopherol) was determined by a modified method of Bai and Gatlin [32]. Pooled larvae (4–5 individuals, ~min 1 g) were homogenized with 4 mL absolute ethanol containing 2% (v/v) pyrogallol and heated at 70°C for 5 min. Subsequently, 1 mL of 60% KOH solution was added, and incubation at 70 °C under nitrogen took place for 20 min, while periodical vigorous shaking of the samples occurred every 5 min. After cooling at room temperature, 3 mL of the organic phase was received and completely dried by nitrogen. A quantity of 1 mL HPLC grade ethanol was added; solutions were vigorously shaken to solubilize the alpha‐tocopherol and subsequent HPLC analysis was carried out. Peaks were detected by a fluorescent spectrophotometer (Waters 474) at an excitation wavelength of 295 nm and an emission wavelength of 335 nm. The column was a µ‐Bondapak 30 cm × 3.9 mm i.d. (Waters Co., USA). The mobile phase was 95% methanol; the flow rate was 2 mL min^−1^ at 23°C. Alpha‐tocopherol standards were used for identification and quantification of vitamin E.

#### 2.3.3. Fatty Acid Determination

The fatty acid composition of the diets and whole fish samples (~2 g) was analyzed by capillary gas chromatography. The lipids were extracted from the samples with chloroform/methanol (2:1, vol by vol), and the extracts purified according to the method by Folch et al. [33]. Total lipid samples were trans‐esterified using methanolic HCl [34] and fatty acid methyl esters (FAME) separated on a fuzed silica capillary column (DB‐225) in an AGILENT (HP) gas chromatograph system (type “6890N”) equipped with flame ionization and mass spectrometer detectors (MSD, type “5973N”). The FAME were identified by using authentic primary (SUPELCO, Bellefonte, PA, USA) or secondary standards (e.g., linseed oil and cod liver oil) and by means of the relationship between the logarithms of relative retention times and chain length of fatty acids. The identity of FAMEs of fatty acids was confirmed by GC–MS runs using NIST Library spectrums or MS spectra of secondary standards. Fatty acid concentrations were expressed as weight percentage of FA, assessed by using the relative response factor and molar concentration of FAME.

### 2.4. Gene Expression of Stress Related Genes

For gene expression studies, around 50 mg of larvae per tank (4–5 individuals) was collected and preserved in RNA stabilization reagent (Sigma, USA) until further use. Total RNA was isolated using SV Total RNA Isolation System (Promega, USA), according to the manufacturer’s instructions. The quality of the total RNA was checked by agarose gel electrophoresis on a 1.5% gel. Total RNA quantity and purity were measured with a NanoDrop 2000 spectrophotometer (Thermo Fisher Scientific, USA). For the qPCR reactions, cDNA was made from mRNA using the iScript cDNA Synthesis Kit (Bio‐Rad, USA). A 1000 ng of total RNA from all samples was used in these reactions, and cDNA was diluted to 10x before measurement. The qPCR reactions were carried out using a Light Cycler 96 instrument (Roche, Switzerland) and a FastStart Essential DNA Green Master Kit (Roche, Germany), which contains SYBR Green as a fluorescent intercalating agent. Genes related to stress and antioxidant defense systems were measured from whole fish, *β-actin* was used as a reference gene. The primers used for the selected genes are presented in Table 6.

**Table 6:** Primers used for real‐time quantitative PCR.

Gene	Forward primer	Reverse primer	GenBank no.	Size (bp)
*sod1*	GACAACACAAACGGCGGCAT	TGGTCCACCGTGAGCTTTATT	NM_131294	69
*gr*	CTGAGACTGCAAGTGTCCAA	CTCTCTCTTCACTATGGCCT	EF042099	156
*hsp70*	CACAATCACCAACGATAAGGG	TTGGCAGACACCTTTTCACGC	JF957366	114
*β-actin*	GCTATGTGGCTCTTGACTTCGA	CCGTCAGGCAGCTCATAGCT	M24113	85

The qPCR reaction solutions consisted of 10 µL Master Mix (2x), 1 µL PCR forward primer (10 µM), 1 µL PCR reverse primer (10 µM), 5 µL cDNA (RT reaction mix), and 3 µL nuclease‐free water. The thermal profile for all reactions was 95°C for 10 min, followed by 40 cycles consisting of 95°C for 10 s, 60°C for 10 s, and 72°C for 10 s. Fluorescence was read at the end of each cycle. The specificity of the reactions was checked by melting curve analysis, and no mispriming or primer dimers were found. All reactions were done in triplicate. The mean threshold cycle (*C*_t_) values were calculated, and the qPCR data were analyzed by the 2^−ΔΔCt^ method [35]. The efficiencies of qPCR reactions were determined using standard curves. Serial dilutions were made from cDNAs of a head kidney and a gill sample. These cDNAs were diluted to 10x, 30x, 90x, 270x, and 810x. Quantitative PCR reactions were carried out on these dilutions with all four primer pairs in triplicate. Standard curves were drawn for each primer pair by plotting *C*_t_ values against the log_10_ of different dilutions of cDNA sample solutions. Efficiencies (*E*) were calculated from the slopes of the standard curves applying the equation *E* = 10^(−1/slope)^. *E* values were 2.05 for *sod1*, 1.80 for *gr*, 2.01 for *hsp70*, and 2.12 for *β-actin*.

### 2.5. Calculations

To determine the growth performance and nutrient utilization of the fish, the following parameters were measured and calculated at the end of the trial:

FBW mg= Final average weight at the end of the experiment,

IBW mg= Initial average weight at the beginning of the experiment.

Specific growth rate (SGR, % day^−1^):

SGR=100×ln FBW−ln IBW/t,

where *t* is the experimental time in days.

Condition factor CF, g cm−3=100×FBW/BL3,

BL mm− body length at the end of trial,

Feed conversion ratio FCR, g g−1= Total amount of feed given g/final total body weight− initial total body weight g,

Protein efficiency ratio PER, g g−1=Final total body weight− initial total body weight g/ total protein consumed g.

### 2.6. Statistical Analysis

Data were analyzed using IBM SPSS Statistics version (29.0.1.0). Normality and homogeneity of variances were verified using the Shapiro–Wilk and Levene’s tests, respectively. Differences among experimental groups were analyzed by one‐way ANOVA followed by Tukey’s post hoc test. Two‐way ANOVA was used to evaluate the effects of ingredient sources, WSVs dose, and their interaction. When significant main or interaction effects were observed (*p* < 0.05), differences among means were further analyzed using Duncan’s multiple range test for post hoc comparisons. For stress‐challenge‐related parameters, a three‐way ANOVA was performed with diet composition, WSVs dose, and sampling point (before stress, immediately after stress, and 4 days after stress) as fixed factors.

The nonparametric Kruskal–Wallis test, followed by Dunn’s post hoc test was used for analyzing the expression of targeted stress‐related genes (*sod1*, *gr*, and *hsp*) because the data did not follow normal distribution. The relationship between vitamin C content of fish body and expression of *hsp70* and *sod1* genes was explored with the computing Pearson correlation. Data are presented as mean ± standard deviation (SD), except for gene expression results, which are expressed as median ± IQR. A significance level of *p*  < 0.05 was used to determine statistically significant differences.

## 3. Results

### 3.1. Developing Weaning Strategy for Better Utilization of Microdiet by Common Carp Larvae

**Figure 3:**
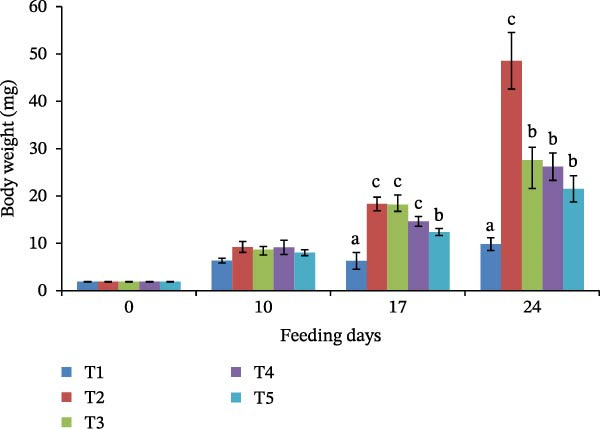
Growth of larvae fed with *Artemia* and dry feed applied in different feeding schedule. Different lowercase letters on the graph bars indicate significant results between the between dietary treatments T1–T5 (*p* < 0.05) at different time of sampling. Data expressed as mean ± SD.

According to the body weight of the fish, the difference was significant after 10 days of feeding between the T1 and the other treatments (Figure 3) and this difference increased over time. The body weight of larvae in this group at final measurement was 9.8 mg compared to fish in T3–T5 treatment ranging between 21.5–27.6 mg. The best‐performing group was T2, where 48.6 mg body weight was achieved. Statistically significant difference in growth parameters appears from day 17 dph between the T2 and the other groups. Differences in feed utilization, protein efficiency ratio, and SGR at the end of feeding were not significant between T3 and T5 groups; however, the highest FCR and lowest PER and SGR in T5 treatment was detected (Table 7). Statistically, better performance based on the investigated parameters for T2 treatment was achieved. For instance, PER ranged between 0.79 and 0.96 in the case of T3–T5, while for T2, 3.50 was calculated.

**Table 7:** Growth performance and nutrient utilization of common carp larvae at the end of trial (at 28 dph) (mean ± SD).

Treatments	T1	T2	T3	T4	T5	*p*‐Value
IBW (mg)	1.89	1.89	1.89	1.89	1.89	—
FBW (mg)	9.84 ± 1.34^a^	48.56 ± 5.97^c^	27.57 ± 2.71^b^	26.20 ± 2.90^b^	21.50 ± 2.77^b^	<0.001
BL (mm)	10.33 ± 0.14^a^	16.70 ± 0.76^c^	13.97 ± 0.51^b^	13.62 ± 0.51^b^	12.80 ± 0.46^b^	<0.001
CF (g cm^−3^)	0.89 ± 0.09^a^	1.04 ± 0.01^b^	1.01 ± 0.02^ab^	1.04 ± 0.06^b^	1.02 ± 0.02^b^	0.024
FCR (g g^−1^)	7.80 ± 1.99^c^	0.82 ± 0.02^a^	1.63 ± 0.51^b^	1.55 ± 0.49^b^	2.26 ± 0.33^b^	<0.001
SGR (% day^−1^)	4.38 ± 0.04^a^	12.95 ± 0.59^c^	9.88 ± 0.19^b^	9.70 ± 0.38^b^	9.19 ± 0.50^b^	<0.001
PER (g g^−1^)	0.24 ± 0.01^a^	3.50 ± 0.49^c^	0.96 ± 0.03^b^	0.89 ± 0.11^b^	0.79 ± 0.08^ab^	<0.001
SR (%)	55.6 ± 7.5^a^	87.4 ± 1.6^c^	73.7 ± 4.2^b^	74.3 ± 1.4^b^	80.2 ± 1.7^bc^	<0.001

*Note*: T1–T5 represent different feeding regime according to Table 1. Statistical *p*‐values refer to differences between treatments T1–T5. Values in the same line with different superscript lowercase letters are significantly different (*p* < 0.05).

Abbreviations: BL, body length; CF, condition factor; FBW, final body weight; FCR, feed conversion rate; IBW, initial body weight; PER, protein efficiency ratio; SGR, specific growth rate; SR, survival rate.

**Figure 4:**
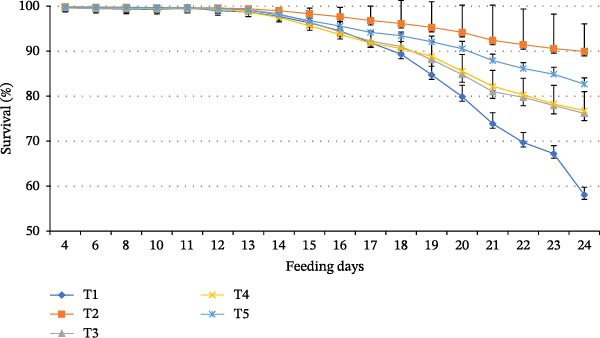
Survival of larvae fed with different feeding schedule. T1–T5 represent different feeding regime according to Table 1. Data expressed as mean ± SD.

Studying the survival rate (Figure 4), differences were shown from the 16^th^ day of feeding. The best survival rate (89.9%) was observed in the group (T2) fed for the longest time with live prey. The lowest survival (58.1%) was found in the “dry feed group” (T1) at the end of the experiment, differing significantly from the rest of the treatments. No significant differences were found between the survival rate of T3–T5 groups, but in T3 and T4 groups, the mortality was significantly higher compared to T2 (Table 7).

The proximate analysis data showed that in the T2 group, fish had the highest and T1 the lowest CP content (Table 8). A significant difference in water content of the larvae was observed between T1 and T2 and between T2 and T5, with the highest level in T1 (86.42%). In respect of fatty acid profile of the larvae, the highest level of saturated fatty acids (SFAs) and lowest level in monounsaturated acids (MUFAs) and polyunsaturated fatty acids (PUFAs) in the T1 group were detected, but these data were not differing significantly. However, in the T2 group, which was fed mostly with *Artemia*, the fish larvae accumulated significantly less eicosapentaenoic acid (EPA), (docosahexaenoic acid (DHA), and arachidonic acid (ARA) compared to other groups. Moreover, T1 fish contained significantly higher ARA than the rest of the treatments.

**Table 8:** Proximate and fatty acid composition of the larvae at the end of trial (at 28 dph).

Treatments	T1	T2	T3	T4	T5	*p*‐Value
Proximate composition (w.w, %)						
Water	86.42^c^	84.20^a^	84.09^a^	84.32^ab^	85.13^bc^	0.001
Crude protein	9.38^a^	12.28^b^	10.74^ab^	11.13^ab^	10.95^ab^	0.050
Crude ash	1.54^a^	1.80^b^	1.76^b^	1.66^ab^	1.55^ab^	0.012
Fatty acid composition (w% FA)						
Total SFA	33.761	28.828	28.650	28.570	29.182	0.354
Total MUFA	21.246	24.430	25.513	24.036	25.349	0.560
Total n‐6	9.819	10.106	12.307	12.900	12.348	0.054
Total n‐3	27.306	29.794	27.765	27.619	27.120	0.246
Total PUFA	37.125	39.900	40.071	40.519	39.468	0.556
EPA + DHA	24.394^b^	16.791^a^	23.866^b^	23.795^b^	23.680^b^	0.004
ARA	3.249^c^	1.488^a^	2.397^b^	2.422^b^	2.520^b^	0.001

*Note*: T1–T5 represents different feeding regime according to Table 1. Statistical *p*‐values refer to differences between treatments T1–T5. Values in the same line with different superscript lowercase letters are significantly different (*p* < 0.05).

Abbreviations: ARA, arachidonic acid; DHA, docosahexaenoic acid; EPA, eicosapentaenoic acid; MUFA, monounsaturated fatty acids; SFA, saturated fatty acids.

**Figure 5:**
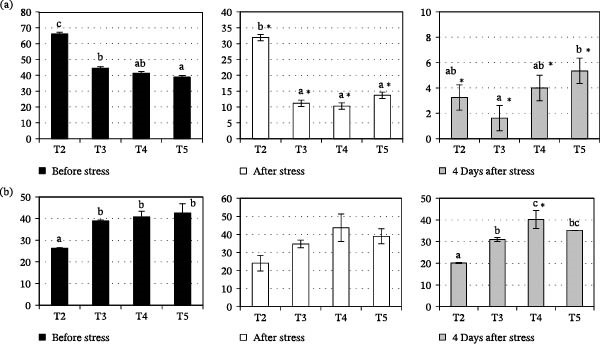
(a) Vitamin C status of carp larvae during the stress experiment. (b) Vitamin E status of carp larvae during the stress experiment. T2–T5 represent different feeding regime according to Table 1. Data expressed (µg g^−1^) as mean ± SD. Different letters indicate significant difference between treatments (T2–T5) following one‐way ANOVA. Asterisk ( ^∗^) indicates significant difference compared to “before stress”.

In the confinement and mild cold stress experiment, significant effects of feeding regime and stress exposure on vitamin C and vitamin E status of larvae were observed (Figure 5). The feeding regime significantly influenced both vitamin C and vitamin E levels (*p* < 0.001). Vitamin C concentrations were generally higher in treatment T2 and lower in T3–T5, whereas vitamin E levels increased from T2 to T4 and remained relatively high in T5 (Table S1).

Stress time also had a significant effect on both vitamins, although the response patterns differed. For vitamin C, concentrations decreased markedly after stress exposure, reaching the lowest values at the recovery time. In contrast, vitamin E showed a more moderate variation across stress periods, with a slight decrease after stress but overall less pronounced changes compared to vitamin C.

A significant interaction between feeding regime and stress time (FR × ST) was observed for vitamin C (*p* < 0.001), indicating that the effect of stress on vitamin C levels depended strongly on the feeding treatment. However, no significant interaction was found for vitamin E (*p* = 0.714), suggesting that its response to stress was largely independent of the feeding regime. Mortalities occurred during the stress challenge, but no differences between groups were detected during this time (data not shown here).      

### 3.2. Development of an Effective Microdiet for Delivering WSVs to Carp Larvae

During 10 days of feeding with microdiets, the body weight of the carp larvae has been doubled in all of the treatments at 26 days after hatching (dph). Significant differences (*p* < 0.05) could be observed between the ingredient sources (plant or fish) on final weight, SGR and feed conversion ratio, but survival (%) of carp larvae was not differing significantly (Table 9). Similarly, different encapsulation materials (nonencapsulated, protein encapsulated and oil encapsulated) did not affect the growth of larvae.

**Table 9:** Production parameters of larvae fed with different with different encapsulation and with different feed ingredient sources microdiets for 10 days (at 26 dph) (mean ± SD).

Diets	IBW (mg)	FBW (mg)	SGR (% day^−1^)	FCR (%)	SR (%)
FB‐CTRL	23.61 ± 2.11	65.01 ± 4.04^b^	10.12 ± 0.62^b^	0.90 ± 0.08^a^	96.5 ± 2.1
PB‐CTRL	23.61 ± 2.11	53.46 ± 8.55^a^	8.11 ± 1.61^a^	1.59 ± 0.76^b^	94.5 ± 4.9
FB‐Pr	23.61 ± 2.11	62.81 ± 6.09^b^	9.76 ± 0.97^b^	0.86 ± 0.18^a^	98.4 ± 1.7
PB‐Pr	23.61 ± 2.11	49.68 ± 0.48^a^	7.44 ± 0.10^a^	1.57 ± 0.11^b^	89.0 ± 1.4
FB‐O	23.61 ± 2.11	57.96 ± 2.86^b^	8.98 ± 0.49^b^	1.23 ± 0.04^a^	88.5 ± 3.5
PB‐O	23.61 ± 2.11	49.61 ± 8.59^a^	7.35 ± 1.74^a^	1.52 ± 0.54^b^	95.0 ± 5.7
Test of between subject effects					
Ingredient (FB–PB)					
FB	—	61.93 ± 4.77	9.62 ± 0.77	1.00 ± 0.20	93.3 ± 4.93
PB	—	50.92 ± 5.77	7.63 ± 1.12	1.56 ± 0.42	92.3 ± 3.50
* p*	—	0.018	0.020	0.047	0.694
Encapsulation material (Pr‐O)					
Contr	—	59.2 ± 8.62	9.12 ± 1.53	1.25 ± 0.59	95.5 ± 3.32
Pr	—	56.2 ± 5.11	8.60 ± 0.95	1.22 ± 0.35	92.0 ± 1.53
O	—	53.8 ± 7.11	8.16 ± 1.40	1.37 ± 0.35	91.0 ± 3.56
* p*	—	0.471	0.511	0.838	0.250
Interaction (diet ingredient × encapsulation material)					
* p*	—	0.848	0.906	0.709	0.167

*Note*: Statistical *p*‐values refer to differences between dietary treatments. Different superscript lowercase letters denote significant differences at *p* < 0.05. FB‐CTRL, fish‐based diet none caps; PB‐CTRL, plant‐based diet none caps; FB‐Pr, fish‐based diet with protein caps; FB‐O, fish‐based diet with oil caps; PB‐Pr, plant‐based diet with protein caps; PB‐O, plant‐based diet with oil caps; Contr, none encapsulation; Pr, protein encapsulation; O, oil encapsulation.

Abbreviations: FBW, final body weight; FCR, feed conversion ratio; IBW, initial body weight; SGR, specific growth rate; SR, survival rate.

### 3.3. Effectiveness of Weaning Diet for Delivery of WSVs

#### 3.3.1. Growth Performance and Nutrient Utilization

**Figure 6:**
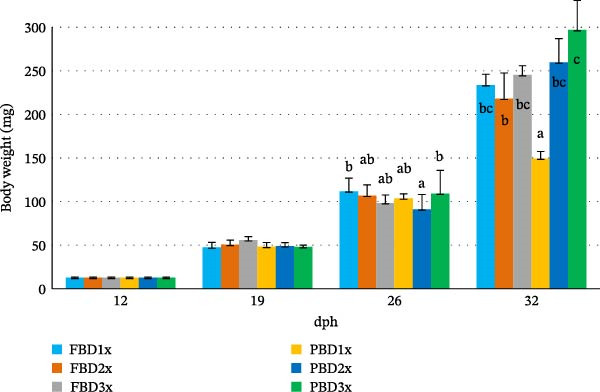
Growth performances of larvae fed with fish‐based (FBD1x–3x) and plant‐based dry feed (PBD1x–3x) from 12 to 32 dph. Different letters on the graph bars indicate significant results between the between dietary treatments FBD1x–3x and PBD1x–3x (*p* < 0.05) at different time using one‐way ANOVA. Data expressed as mean ± SD.

**Figure 7:**
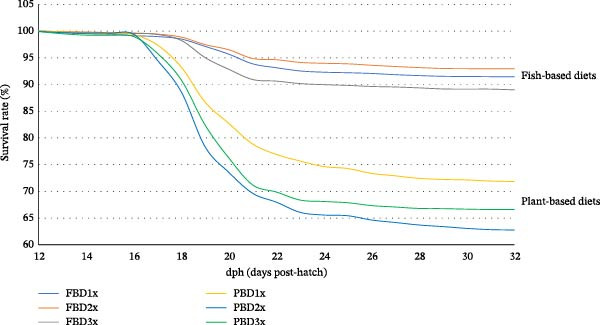
Survival (%) of larvae fed with fish (FBD1x, FBD2x, and FBD3x) and plant based dry feed (PBD1x, PBD2x, and PBD3x) from 12 to 32 dph.

The growth performance of the larvae was within a favorable range (150–300 mg) at the end of the feeding trial, and well‐developed bodies were observed in all groups (Figure 6). Significant differences between the fish‐ and plant‐based dietary groups were detected with respect to SGR (SGR) (Table 10) and survival rate, both being more pronounced in the groups fed fish‐based diets (Figure 7). However, due to the lower number of fish individuals in the plant‐based dietary groups, a noticeably higher final body weight (FBW) was observed in these groups (Figure 6). In addition, the lowest condition factor (CF) and SGR were measured in the PBD1x group. Survival rates in the plant‐based diet groups fluctuated between 41.0% and 83.0%, whereas in the fish‐based diet groups, the range was 89.0%–91.4%. Significant differences in FBW, SGR, and CF were also observed among the different vitamin dosage groups (Table 10). Fish fed diets with the highest WSV content exhibited more intensive growth performance compared to fish fed diets with lower vitamin levels. Moreover, significant interactions between the ingredient type and vitamin dose were determined with respect to SGR and FBW. Unfortunately, the feed conversion rate and nutrient utilization were not determined. It was found that the CP and crude ash contents of the fresh larvae did not differ among treatments (Table 10). However, the water content of larvae in the fish‐based dietary groups was significantly higher than that in the plant‐based dietary groups.

**Table 10:** Growth performance and body composition (w.w.) of carp larvae at the end of feeding period (mean ± SD).

Diets	IBW (mg)	FBW (mg)	CF (g cm ^−3^)	SGR (% day^−1^)	SR (%)	Water (%)	Crude protein (%)	Crude ash (%)
FBD1x	12.68	233.7 ± 52.5^bc^	1.73 ± 0.04	12.8 ± 0.47^b^	91.4 ± 2.20^c^	82.6 ± 0.04^b^	12.0 ± 0.18	2.04 ± 0.06
FBD2x	12.68	218.1 ± 57.7^b^	1.86 ± 0.09	12.6 ± 0.28^b^	92.9 ± 0.55^c^	82.5 ± 0.37^b^	11.9 ± 0.11	1.95 ± 0.01
FBD3x	12.68	245.3 ± 62.9^bc^	1.79 ± 0.08	13.4 ± 0.27^b^	89.0 ± 4.47^bc^	82.7 ± 0.34^b^	11.9 ± 0.02	1.96 ± 0.00
PBD1x	12.68	149.4 ± 73.5^a^	1.76 ± 0.09	9.88 ± 0.45^a^	71.8 ± 3.31^abc^	81.3 ± 0.54^a^	11.8 ± 0.03	2.29 ± 1.28
PBD2x	12.68	276.7 ± 32.8^bc^	1.96 ± 0.13	12.7 ± 0.48^b^	60.2 ± 17.5^a^	81.3 ± 0.46^a^	12.1 ± 0.05	1.82 ± 0.05
PBD3x	12.68	296.8 ± 33.9^c^	1.97 ± 0.13	12.9 ± 0.81^b^	66.6 ± 9.23^ab^	81.3 ± 0.08^a^	11.9 ± 0.53	1.83 ± 0.01
*p*	—	<0.001	0.047	0.006	0.001	<0.001	0.725	0.679
Ingredients (I)								
FBD	—	232.4 ± 20.5	1.80 ± 0.1	12.9 ± 0.5	91.1 ± 3.0	82.6 ± 0.3	11.9 ± 0.1	2.0 ± 0.1
PBD	—	241.0 ± 73.5	1.90 ± 0.1	11.8 ± 1.8	66.2 ± 11.2	81.3 ± 0.4	11.9 ± 0.3	2.0 ± 0.5
* p*	—	0.468	0.114	0.021	<0.001	<0.001	0.803	0.951
Vitamin dosage (VD)								
1x	—	191.6 ± 47.1	1.75 ± 0.07	11.3 ± 1.71	81.6 ± 11.0	82.0 ± 0.8	11.9 ± 0.2	2.2 ± 0.6
2x	—	247.4 ± 43.3	1.91 ± 0.11	12.7 ± 1.14	76.6 ± 21.1	81.9 ± 0.8	12.0 ± 0.2	1.9 ± 0.1
3x	—	271.0 ± 36.1	1.88 ± 0.13	13.1 ± 0.60	77.8 ± 13.9	82.0 ± 0.8	11.9 ± 0.3	1.9 ± 0.1
* p*	—	<0.001	0.037	0.012	0.571	0.916	0.708	0.435
Interaction (I × VD)								
* p*		<0.001	0.569	0.034	0.394	0.916	0.419	0.630

*Note*: FBD1x, FBD2x, and FBD3x, fish‐based diets. PBD1x, PBD2x, and PBD3x, plant‐based diets. Statistical *p*‐values refer to differences between treatments using one‐way ANOVA and values in the same row with different superscript lowercase letters are significantly different (*p* < 0.05). Differences between ingredients, between vitamin level, and interaction between the ingredient and vitamin dosage are presented following two‐way ANOVA.

Abbreviations: CF, condition factor; FBW, final body weight; IBW, initial body weight; SGR, specific growth rate.

#### 3.3.2. Delivery of Vitamins

The retention of vitamins in common carp larvae following feeding with vitamin‐enriched diets seems to be correlated with the dietary ingredient sources (fish‐based or plant‐based diet; Table 11). However, significant difference in vitamin B_6_ and B_1_ content was not determined; meanwhile, the vitamin C content in larvae fed with fish‐based diets was significantly lower and vitamin E content was significantly higher compared to those in the plant‐based dietary groups. Surprisingly, the vitamin concentrations did not directly reflect the vitamin content of the diets but were more closely correlated with the larvae’s growth and nutrient uptake.

**Table 11:** Vitamin concentration (w.w.) at the end of feeding trial with common carp larvae fed with fish‐based diets (FBD1x, FBD2x, and FBD3x) and plant‐based diets (PBD1x, PBD2x, and PBD3x).

Diets/vitamin	B_6_	C	E	B_1_
	(µg g^−1^) (mean ± SD)			
FBD1x	33.2 ± 23.0^ab^	12.2 ± 1.17^ac^	24.3 ± 1.30^c^	10.6 ± 4.87
FBD2x	40.5 ± 30.1^b^	8.09 ± 4.82^a^	24.6 ± 2.56^c^	10.9 ± 4.99
FBD3x	31.8 ± 19.8 ^ab^	20.2 ± 6.27^ab^	21.9 ± 1.47^bc^	12.7 ± 5.95
PBD1x	27.3 ± 5.07^ab^	26.8 ± 1.35^bc^	17.1 ± 0.19^ab^	14.7 ± 0.95
PBD2x	10.2 ± 6.64^a^	27.0 ± 13.5^b^	11.8 ± 2.95^a^	13.7 ± 0.62
PBD3x	21.6 ± 6.36^ab^	22.9 ± 3.69^bc^	11.0 ± 1.42^a^	14.3 ± 0.82
*p*	<0.031	0.003	<0.001	0.075
Ingredient (I)				
FBD	35.1 ± 21.7	13.5 ± 6.7	23.6 ± 2.1	11.4 ± 4.7
PBD	19.7 ± 9.2	25.6 ± 7.3	13.3 ± 3.3	14.2 ± 0.8
* p*	0.093	<0.002	<0.001	0.138
Vitamin dosage (VD)				
1x	30.2 ± 15.2	19.5 ± 8.09	20.7 ± 4.05	12.6 ± 3.86
2x	25.3 ± 25.6	17.6 ± 13.8	18.2 ± 7.44	12.3 ± 3.54
3x	26.7 ± 14.3	21.6 ± 4.81	16.5 ± 6.09	13.5 ± 3.90
* p*	0.888	0.591	0.106	0.865
Interaction *p* (I × VD)	0.476	0.132	0.071	0.847

*Note*: Statistical *p*‐values refer to differences between treatments using one‐way ANOVA and values in the same row with different superscript lowercase letters are significantly different (*p* < 0.05). Differences between ingredients, between vitamin level, and interaction between the ingredient and vitamin dosage are presented following two‐way ANOVA.

#### 3.3.3. Stress Challenge Tests

##### 3.3.3.1. Confinement Stress

**Figure 8:**
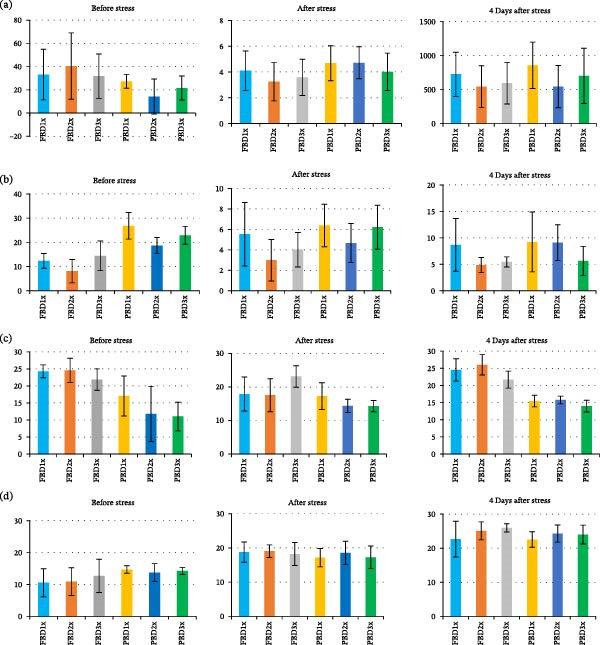
Vitamin concentrations (w.w) in the confinement stress experiment with common carp larvae fed with fish‐based (PBD1x, PBD2x, and PBD3x) and plant‐based (PBD1x, PBD2x, and PBD3x) diets. Data expressed (µg g^−1^) as mean ± SD. (a) Vitamin B_6_. (b) Vitamin C. (c) Vitamin E. (d) Vitamin B_1_.

Under confinement stress (Figure 8), a significant effect of dietary ingredient source on the antioxidant vitamin status of larvae was detected (Table S2, ingredients). In contrast, dietary vitamin enrichment levels did not significantly affect vitamin concentrations in fish larvae (Table S2, vitamin dosage).

Stress time had a marked effect on vitamin status. Vitamin B_6_ concentrations significantly decreased (*p* < 0.001) following stress in all groups; however, after the recovery period, at 4 days post‐stress, vitamin B_6_ levels increased drastically in the larvae (Figure 8a). Similarly, vitamin C levels were depleted after stress and remained significantly lower even 4 days post‐stress (Figure 8b). In the case of vitamin E, stress time also significantly affected larval vitamin E content (*p* < 0.040) (Figure 8c). Vitamin B_1_ showed an increase in response to stress (Figure 8d), with a further significant elevation detected at 4 days post‐stress (Table S2, stress time). Additionally, significant interactions were observed between stress time and dietary sources for vitamins C, E, and B_1_, but not for dosage of vitamin.

**Figure 9:**
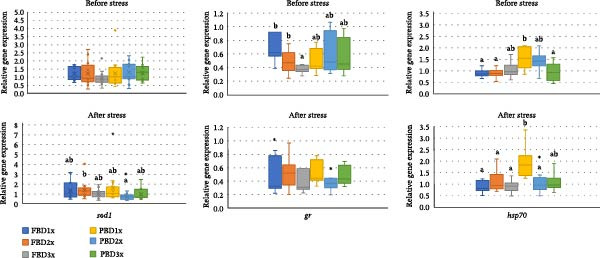
Changes in relative expression of stress‐related genes (*sod1*, *gr*, and *hsp70*) between different treatments (FBD1x, FBD2x, FBD3x, PBD1x, PBD2x, and PBD3x) and in stressed larvae compared to nonstressed ones in groups. Different lowercase letters indicate significant difference between treatments using one‐way ANOVA. Asterisk ( ^∗^) indicates significant difference to before stress. Data expressed as median ± IQR.

The expression levels of superoxide dismutase‐1 (*sod1*), glucocorticoid receptor (*gr*), and heat shock protein 70 (*hsp70*) genes were evaluated before and after confinement stress exposure. Following the stress challenge, *sod1* expression was significantly reduced in the PBD2x group compared to the corresponding pre‐stress value and compared to the FBD2x treatment (Figure 9); however, neither dietary ingredient source nor stress duration exerted a significant overall effect on *sod1* expression according to the statistical analysis (Table S3).

A similarly inconsistent pattern was observed for *gr* expression. Significant downregulation was detected in the FBD1x and PBD2x groups after stress exposure relative to pre‐stress levels, while significant differences were also identified between the FBD1x and FBD3x groups prior to stress. Nevertheless, two‐way ANOVA revealed no significant main effects of dietary ingredient source, vitamin dosage, or stress duration on *gr* expression (Table S3).

In contrast, the relative expression of *hsp70* was significantly influenced by the dietary ingredient source, vitamin dosage, and their interaction but not by the stress time. Specifically, the *hsp70* expression was significantly downregulated in the PBD2x group following stress exposure. Moreover, the plant‐based diet with the lower vitamin supplementation level (PBD1x) exhibited significantly elevated *hsp70* expression compared to the other dietary groups, both before and after stress exposure (Figure 9).

##### 3.3.3.2. Hypoxia Stress

**Figure 10:**
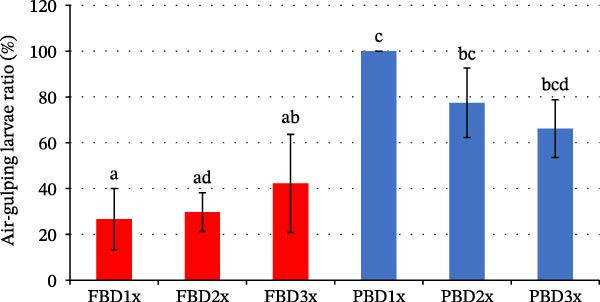
Average of air‐gulping larvae (%) at the end of hypoxia stress. FBD, fish‐based diet; PBD, plant‐based diet. Different letters on the graph bars indicate significant results between the between dietary treatments (*p* < 0.05). Data expressed as mean ± SD.

The results on number of air‐gulping larvae following the hypoxia stress are more attractive, hence deviation to stress resistance of fish fed with different feed ingredients could be observed (Figure 10). In this sense the number of air‐gulping larvae in groups fed with fish‐based diets was much lower (30%–40%) compared to the groups fed with plant‐based feeds (60%–100%) (*p* < 0.001). The highest fish ratio in group PBD1x was found and significantly differed from larvae in FBD groups. Additionally, dose of vitamin was not generally affected the larvae performance in stress situation (*p* = 0.404), while the interaction effect between ingredient origin and vitamin dosage exhibited a significant effect (*p* = 0.026).

## 4. Discussion

Due to their rapid growth and continuous feeding, fish larvae require a high total nutrient intake and are highly sensitive to stress, necessitating specific biotic and abiotic conditions for proper survival, development, and growth [36]. The nutrient content of *Artemia* nauplii is at its highest level immediately after hatching, making it easily utilizable by carp larvae. In the first trial, the best performing group was T2, where feeding for 24 days was performed with *Artemia*. The treatments with different co‐feeding periods with *Artemia* (T3–T5) performed much better compared to T1 but worse compared to T2. Data on growth performance and nutrient utilization clearly indicated that 7–8 days of feeding with live prey feed is the minimum required period for survival and better growth. In the early weaning group (T1), the survival rate was the lowest; however, significant differences compared to the T2 group can be seen from 8 days past the last *Artemia* feeding (among 16 and 24 days of feeding; Figure 4). This result indicates that carp larvae at the age of 8 dph can store essential nutrients from *Artemia* co‐feeding for more than 1 week, resulting in a delay in mortality. This mortality is caused by the immature digestive system of the larvae, which could either not accept or digest the new microparticulate diet and have not started feeding on that. The body composition data of whole larvae similarly proves that *Artemia* is a good source of digestible protein from the first feeding of carp compared to microdiet. This is confirmed by studies performed on a wide range of fish species, including common carp [7, 37, 38].

The fatty acid profile of larvae fed with microdiet based on marine origin mirrored the composition of the diets, in this sense contained a higher amount of long‐chain PUFA (Lc‐PUFA) compared to T2. However, this more affordable fatty acid profile in the body did not affect the growth and survival rate of fish in this stage. The significantly higher survival rate in the T5 group compared to those in T3 and T4 may be due to the longer duration of microdiet application in the T5 group’s feeding regime. This suggests that once the digestive system of carp larvae is sufficiently developed to utilize hydrolyzed protein, it is more beneficial to replace *Artemia* with high‐quality and nutritious microdiets. The highest vitamin C level (Figure 5a) observed in the control group (T2) at the end of the feeding period demonstrated intensive vitamin C uptake from live feed, as was similarly observed by Yufera et al. [22] or Dabrowski [39]. As the *Artemia* feeding period decreased, the vitamin C level in the larvae’s bodies declined, while the vitamin E level increased due to the higher bioavailability of vitamin E in the dry feed compared to *Artemia*. This suggests that the vitamin C supplementation level in the microdiet was the minimum required for carp larvae, likely due to its intensive utilization by the fish. Vitamin C and E are recognized as potent antioxidants because they effectively capture peroxyl radicals in the aqueous phase, preventing the initiation of lipid peroxidation and thereby protecting cell membranes [40, 41]. During the stress situation administered at the end of our trial (28 dph), the utilization of vitamin C and E differed. Vitamin C levels dropped drastically to a low level across all groups, indicating that fish consumed most of their vitamin C to cope with the stress, regardless of their initial vitamin status. In contrast, vitamin E was less consumed, showing a vitamin E‐sparing, vitamin C‐consuming effect during the stress event. Nevertheless, in groups T2, T3, and T5, the larvae used approximately 8–10 µg g^−1^ (w.w.) of vitamin E to manage the stress conditions. The sparing effects between vitamin C and E were already demonstrated by several studies [42–44] when the function of the antioxidant defense system in fish was evaluated. We concluded based on the data on production parameters that feeding common carp larvae with *Artemia* for 8 days, followed by a 4‐day co‐feeding period, is necessary to initiate the utilization and nutrient uptake from microdiets. The vitamin C status of larvae was almost depleted during the stress event in larvae with shorter *Artemia* feeding and still were not replenished by an inert diet during the recovery period. These results suggest that dietary composition played an important role in the modulation of vitamin C metabolism during stress, whereas vitamin E homeostasis appeared to be less affected by the feeding regime.

In experimental trial 2, different sources of ingredients and encapsulation matrices were investigated, and it was observed that performance of fish fed with marine ingredients was better compared to dietary groups fed with plant sources. It means that replacing the fish sources with plants will significantly decrease the growth and survival rate and nutrient utilization of the carp larvae. The principal question is obviously whether larval fish are capable of utilize these microdiets. Replacement of marine ingredients with plant sources in the larval diet and promoting earlier weaning have been set as objectives behind the use of microdiets [8, 23]. Diets with enhanced digestibility are more likely to meet the nutritional requirements of larvae and, consequently, are expected to improve both growth performance and survival rates. It seems that the plant proteins investigated in this study (soy protein isolate, pea protein concentrate, and wheat gluten) and the diet formulated using the given mixing rate (Table 2) were not digested efficiently and utilized by carp larvae, in contrast with the study of Cahu et al. [23]. However, the diets differed in several aspects beyond the protein source itself. Marine oils were replaced with plant oil, which does not provide Lc‐PUFA, such as DHA and EPA, in the same way as fish oil or krill oil. Such a deficiency alone may be sufficient to compromise larval growth and survival [45, 46]. In addition, fish meal may contain marine‐derived feed attractants, whereas plant meal lacks these components. Considering these factors, the observed differences in larval performance between the fish‐based and plant‐based dietary groups may have been related more to the overall ingredient origin than specifically to the protein source itself. Therefore, the diets formulated in the present experiment demonstrated the inevitable use of marine sources for the early feeding of larvae. Additionally, the larvae performance by using fish protein hydrolysates may allow us to remove the live food after pre‐condition of larvae with co‐feeding protocols with *Artemia* up to 12 pdh, as was already demonstrated in the first trial. Regarding the encapsulation of WSV, either protein matrix or oil matrix allows encapsulation without detrimental effect to the production parameters.

The main goal of experimental trial 3 was to investigate the WSV retention during the feeding period and to assess the utilization of the antioxidant vitamins by larvae (32 pdh) during confinement stress, quite in a similar way, as was performed in the first trial. Besides vitamin C and E, two vitamin B, thiamin (B_1_) and pyridoxine (B_6_), were assessed to follow up the vitamin requirement and their role in the stress defense pathway. Parallel with confinement stress, hypoxia stress was administered and followed the reaction of the larvae to the low oxygen exposure. Based on the production parameters assessed at the end of this feeding experiment, a low survival rate was observed in plant‐based dietary groups. Survivals of fish in FBD groups (89%–92%) were comparable with the results described by Dabrowski et al. [47]. Due to the higher mortality in PBD dietary groups, the FBW of the remaining population became higher compared to the groups fed with fish‐based diets. Under these circumstances, the performance of the fish during the trial was strongly influenced by the varying stocking densities in the tanks. In addition, the vitamin content of the diet had a significant positive effect on larval growth, and this effect also depended on the dietary source. This indicates that vitamin supplementation had a more pronounced effect on growth in larvae fed fish‐based diets compared to plant‐based diets.

The origin of the diets had an impact only on the vitamin C and E status of the larvae following the feeding trial, while both types of feed may provide similar levels of B_1_ and B_6_. Larvae fed plant‐based diets showed higher vitamin C incorporation, whereas those fed fish‐based diets exhibited significantly higher vitamin E levels. This observation aligns with previous findings indicating that plant‐derived ingredients may contribute to improved vitamin C retention capacity in fish due to their naturally occurring bioflavonoids [39, 48]. Regarding vitamin enrichment dosages, no differences were observed among the treatment groups. This may highlight that either a higher dose of certain vitamins than required could not be effectively retained by the larvae or leaching out of WSVs from the tested encapsulated microdiets. One of the most complicated technical problems to solve in the development of formulated larval feeds is to prevent the high rate of leaching of hydrosoluble compounds after the rehydration of the particles during the supply to rearing tanks. The leaching of hydrosoluble components such as vitamins has been studied in different types of microdiets, showing high differences among the types of microdiets [49, 50]. The encapsulation method tested in this trial to deliver WSV did not increase the vitamin content in the fish body following the feeding period. Nevertheless, the B_1_ and B_6_ levels detected in all of the groups satisfy the requirement of carp larvae for proper development (0.5 mg kg^−1^ B_1_ and 5 mg kg^−1^ B_6_) according to NRC 2011 [48].

Following the stress experiments, effects of diet composition on larvae vitamin utilization were experienced. There was no observed mortality during the confinement stress. In addition, the antioxidant vitamins C and E, were consumed to protect against the stress with a sparing effect between vitamins, as was seen in the first experiment. Increasing the B_1_ and B_6_ vitamin levels in the body of carp larvae during confinement stress and after a 4‐day post stress period was observed. In general, there is no information on diseases related to vitamin B deficiency and stress‐induced lesions, making these data particularly important. In our previous study with carp larvae, decrease of vitamin B_1_ was found after stress and after 4‐days post stress, as long as vitamin B_6_ increases [51], which is similar to the findings of this study. Similar findings were reported by Kumar et al. [52] in milkfish (*Chanoschanos*) and Zhang et al. [53] in largemouth bass (*Micropterus salmoides*) following fortification of the diet with vitamin B_6_. The increase in vitamins B_1_ and B_6_ following confinement stress may indicate metabolic adjustments associated with the stress response rather than a direct increase in nutritional demand. However, this increase may indicate the need to enhance vitamin B_6_ supply to protect against protein degradation and increase transaminase activity in the later stages of development [54]. Both vitamins act as essential cofactors in metabolic pathways that are closely linked to cellular stress tolerance. B_1_ contributes to carbohydrate metabolism and redox homeostasis through its role in the pentose phosphate pathway [55], while B_6_ is involved in amino acid metabolism and antioxidant defense mechanisms [53, 54]. The elevated levels observed in stressed larvae may therefore reflect the mobilization or accumulation of these vitamins to support stress‐related metabolic processes. Alternatively, reduced flux through standard metabolic pathways during confinement stress may temporarily decrease their utilization, leading to higher detectable concentrations.

During hypoxia stress, the number of air‐gulping larvae in the FBD groups was considerably lower than that in the PBD groups, with no apparent effect of vitamin supplementation (Figure 10). Overall, these results do not provide strong evidence that dietary vitamin enrichment improves the stress tolerance of common carp larvae. Therefore, it can be concluded that even NRC‐recommended levels of vitamins in fish‐based feeds are sufficient to produce high‐quality carp fry. The reduced hypoxia tolerance observed in larvae fed plant‐based diets is likely the result of several interacting nutritional factors rather than digestibility alone. From our opinion, the strongest explanation is the fatty acid profile of the diets. Plant‐based ingredients generally lack long‐chain n−3 PUFA, such as DHA and EPA, which play important roles in maintaining membrane fluidity, cellular integrity, and physiological responses to stress. A deficiency of these fatty acids may impair metabolic and cardiovascular performance under hypoxic conditions: AA is an eicosanoid precursor, a substrate for prostaglandins, leukotrienes, and thromboxanes, which regulate inflammation, immune response, cardiovascular function, and stress adaptation. Adequate AA is, therefore, important for larval resilience under environmental or nutritional stress. In respect of the lack of essential amino acids, we consider that the microdiets were formulated to contain balanced amino acid content. Furthermore, it contains more digestible proteins as soy protein isolate; however, the plant protein concentrates probably were less digestible by larvae with an immature digestive system compared to CPSP 90 and micro ionized fish meal.

In the confinement stress experiment, several stress‐related gene expressions were assessed and correlated to the vitamin status of larvae. Handling stress in fish (such as netting, crowding, transport, or physical disturbance) triggers a physiological stress response, which typically includes oxidative stress, cellular stress, and activation of the hypothalamic–pituitary–interrenal (HPI) axis (the fish version of the HPA axis) [56]. Superoxide dismutase 1, *sod1*, expression typically indicates a cellular response to oxidative stress. Cells may upregulate *sod1* to protect against damage from reactive oxygen species (ROS), especially under stress conditions like UV light, pollution, toxins, or inflammation [57]. The glucocorticoid receptor (encoded by the *gr* gene) is a nuclear hormone receptor that binds cortisol, the primary stress hormone in fish (produced via the HPI axis). After binding cortisol, the *gr* translocates to the nucleus and regulates the expression of stress‐response genes (e.g., immune, metabolic, and anti‐inflammatory genes) [58]. Higher gene expression typically reflects a physiological or psychological stress response. On the other hand, it can be a marker of chronic or acute stress, regulating downstream genes involved in energy metabolism, immune function, and behavior. Heat shock protein 70 (*hsp70*) is a molecular chaperone that helps refold misfolded proteins and prevents aggregation [59]. It is highly conserved and plays a role in protein homeostasis (proteostasis). The expression of *hsp70* increases in response to stress, such as heat shock and inflammation. Its upregulation is a marker of cellular stress and damage aimed at protecting and stabilizing proteins. It also has roles in immune responses and cell survival. Following 20 days of feeding with microdiets, no significant differences between dietary treatments could be detected in the expression of *sod1*, which means even the type of ingredients or level of vitamins did not influence the antioxidant response of the fish. With respect to the influence of crowded state, which may cause stress to fish, *sod1* expression was suppressed in fish fed diet PBD2x after stress. Contrary to this, a slight upregulation in the FBD treatments compared to pre‐stress values could be detected, allowing for a modest antioxidant response after stress and suggesting some protection. These findings support the hypothesis that fish‐based microdiets may enhance cellular responses to oxidative stress, although no observable impact of dietary vitamin C levels was detected in this case.

The increased expression of *gr* presented by FBD1x and PBD2x groups compared to the rest of the treatments may reflect that the fish were chronically stressed before the confinement occurred. Downregulation after stress (in FBD1x and PBD2x) may mean fish reached a peak stress state before confinement, and the system shut down or reduced expression as a protective measure. However, there is no clear explanation for the chronic stress observed in these groups. In the remaining treatments, a mild, nonsignificant increase following stress suggests a moderate and balanced stress response. In FBD treatments, minimal stress response was found due to *hsp70* not being strongly induced. This means that the fish suffered a mild stress or well‐buffered it. In PBD1x fish, the highest expression was observed before and after stress as well. This suggests a pre‐activation or high cellular defense level maintenance. The significant drop of *hsp70* after confinement stress in the PBD2x group could mean stress overwhelmed the system, the diet lacks support for protein repair, or *hsp70* was downregulated as late‐stage feedback or due to nutrient depletion. This indicates poor cellular stress buffering.

Summarizing the findings, it looks that PBD1x treatment presented the best diet for confinement stress protection, with high *sod1* (strong antioxidant activation post‐stress → protects against ROS), very high *hsp70* (maintains protein integrity → robust cellular resilience), and balanced *gr* (moderate HPI axis response → appropriate hormonal stress regulation without overstimulated or suppressed). PBD1x appears to precondition the fish, likely with ingredients that enhance systemic resilience (possibly vitamin C/E). In this context, considering the vitamin C level in the fish body after feeding (Figure 8), a significantly higher concentration was observed in the PBD1x group compared to FBD2x and PBD2x. However, this increase was accompanied by a reduction in the body weight and other biometric traits in the same group. Nevertheless, the vitamin C level measured in the fish body was not aligned with dietary vitamin C concentrations (diets FBD3x and PBD3x were supplemented with the highest vitamin C, refer to Table 4). Pearson correlation analysis between vitamin C levels in the fish body and the expression of the *hsp70* and *sod1* genes before and after stress event revealed a weak positive correlation. On the other side, between *hsp70* and *sod1* genes, a strong positive correlation was shown before stress (*R* = 0.615, *p* < 0.001) and after stress (*R* = 0.729, *p* < 0.001), respectively. According to these findings, the PBD1x diet exhibited strong antioxidant, cellular stress, and hormonal regulatory effects. However, despite the lower body weight, fish in this group maintained significantly better vitamin C homeostasis and appeared to cope with confinement stress more effectively. In comparison, PBD2x fish failed in the stress defense.

## 5. Conclusions

The feeding trials demonstrated that formulated microencapsulated diets can be effective starter feeds for common carp larvae but only when combined with an optimized weaning strategy. A minimum of 8 days of live prey feeding is required to ensure survival, optimal growth, and stress tolerance. Larval survival was strongly influenced by the origin of the ingredients, with fish‐based diets outperforming plant‐based diets, while the choice of encapsulation material had little effect on growth or nutrient utilization. Vitamin retention from the tested microdiets was limited, indicating a need for improved encapsulation of WSVs. However, the adsorption of vitamin C was much pronounced from plant‐based diet compared to fish‐based diet, whereas the opposite trend was observed for vitamin E. Hypoxia challenge tests demonstrated reduced stress tolerance in larvae receiving plant‐based diets, while higher vitamin C levels in that fish could be connected to enhance the stress resilience during confinement stress. In addition, marked alterations in vitamin B_1_ and B_6_ status were observed in response to stress events in larvae. These findings highlight the importance of combining an effective weaning protocol, marine protein sources, and targeted vitamin C supplementation to maximize survival, growth, and stress tolerance in first‐feeding carp larvae.

## Author Contributions

**Zsuzsanna J. Sándor**: data collection, data analysis and interpretation, drafting manuscript. **László Ardó**: sample collection, gene expression analysis. **Jelena Stanivuk**: formal analysis, statistics. **Attila Terhes**: animal husbandry. **Shivendra Kumar:** conceptualization, data analysis, reviewing and editing.

## Funding

The financial support for this work from the EU Seventh Framework Programme by the ARRAINA Project (Project Number 288925, Advanced Research Initiatives for Nutrition & Aquaculture is gratefully acknowledged. The work of Zsuzsanna J. Sándor was supported by the Research Excellence Programme of the Hungarian University of Agriculture and Life Sciences.

## Conflicts of Interest

The authors declare no conflicts of interest.

## Supporting Information

Additional supporting information can be found online in the Supporting Information section.

## Supporting information


**Supporting Information** Table S1. Effect of feeding regime and stress time on the concentrations of vitamins C and E (cited in Section 3.1). Table S2. Effects of dietary ingredient source and encapsulated vitamin dose on vitamin concentrations in carp larvae following confinement stress (cited in Section 3.3.3). Table S3. Relative expression of stress‐related genes (*sod1*, *gr*, and *hsp70*) at the end of the confinement stress in fish larvae fed with diets differing in ingredients and vitamin dosage (cited in Section 3.3.3).

## Data Availability

The datasets generated during and/or analyzed during the current study are available from the corresponding author upon reasonable request.
