# Supplementary material for: Optimizing Feeding Regimes and Vitamin Delivery Methods in Microdiet for Improving Survival and Growth of Carp Larvae
Source: Aquac Nutr. 2026 Jun 29;2026:3026254. doi: 10.1155/anu/3026254 (PMC13315130; doi:10.1155/anu/3026254)
Supplement: Supplementary file 1 — Supporting Information Table S1. Effect of feeding regime and stress time on the concentrations of vitamins C and E (cited in Section 3.1). Table S2. Effects of dietary ingredient source and encapsulated vitamin dose on vitamin concentrations in carp larvae following confinement stress (cited in Section 3.3.3). Table S3. Relative expression of stress‐related genes (sod1, gr, and hsp70) at the end of the confinement stress in fish larvae fed with diets differing in ingredients and vitamin dosage (cited in Section 3.3.3). [file ANU-2026-3026254-s001.docx]

Supplementary material

**Optimizing feeding regimes and vitamin delivery methods in microdiet for improving survival and growth of carp larvae**

**Table S1**. Effect of feeding regime and stress time on the concentrations of vitamins C and E

| **Vitamin** | **C** | **E** |
| --- | --- | --- |
|  | (µg/g) (mean ± SD) | |
| **Test of between subject effects** |  | |
| **Feeding regime (FR)** |  |  |
| **T2** | 29.4±28.2 | 23.5±3.41 |
| **T3** | 16.6±19.6 | 34.9±3.70 |
| **T4** | 16.5±17.6 | 41.5±4.39 |
| **T5** | 17.3±15.3 | 38.9±4.25 |
| *p* | ***<0.001*** | ***<0.001*** |
| **Stress time (ST)** |  |  |
| BS | 47.7±11.6 | 37.1±7.06 |
| AS | 16.8±9.57 | 35.4±8.60 |
| 4A | 3.56±1.75 | 31.6±8.07 |
| *p* | ***<0.001*** | ***0.023*** |
| **Interaction *p* (FR*ST)** | ***<0.001*** | *0.714* |

BS- before stress, AS-after stress, 4A-4-day after stress. T2-T5 represent different feeding regime according to Table 1. Differences between feeding regime and stress time, and interaction between them are presented following Two-way ANOVA.

**Table S2.** Effects of dietary ingredient source and encapsulated vitamin dose on vitamin concentrations in carp larvae following confinement stress

| **Vitamin** | **B_6_** | **C** | **E** | **B_1_** |
| --- | --- | --- | --- | --- |
|  | (µg/g) (mean ± SD) | | | |
| **Ingredients (I)** *p* | *0.381* | ***<0.001*** | ***<0.001*** | *0.718* |
| **FBD** | 220.2±112.2 | 7.93±3.90 | 22.4 ±3.39 | 18.2±3.47 |
| **PBD** | 241.7±120.8 | 12.9±5.18 | 14.6±3.37 | 18.5±2.45 |
| **Vitamine dosage (VD)** *p* | *0.145* | *0.310* | *0.407* | *0.086* |
| **1x** | 275.4±116.1 | 11.37±4.10 | 19.42±3.63 | 17.69±3.12 |
| **2x** | 191.0±109.4 | 9.27±5.32 | 18.35±3.77 | 18.6±2.92 |
| **3x** | 226.4±124.0 | 10.54±4.19 | 17.67±2.73 | 18.73±2.83 |
| **Stress time (ST)** *p* | ***<0.001*** | ***<0.001*** | ***0.040*** | ***<0.001*** |
| ***BS*** | 27.3±15.7 | 19.6±7.91 | 18.4±4.45 | 12.8±3.18 |
| **AS** | 4.05±1.41 | 4.58±2.48 | 17.4±3.47 | 18.2±2.93 |
| **4AS** | 661.5±332.4 | 7.03±3.22 | 19.6±2.21 | 24.0±2.77 |
| **Interactions** |  |  |  |  |
| **I*VD** *p* | *0.471* | *0.135* | *0.057* | *0.631* |
| **I*ST** *p* | *0.663* | ***0.001*** | ***0.014*** | ***0.002*** |
| **VD*ST** *p* | *0.089* | *0.675* | *0.011* | *0.725* |
| **I*VD*ST** *p* | *0.859* | *0.206* | *0.231* | *0.687* |

FBD-fish-based diet, PBD-plant-based diet, BS-before stress, AS-after stress, 4AS-4-day after stress

I*VD- ingredient*vitamin dosage, I*ST-ingredient*stress time, VD*ST- vitamin dosage*stress time, I*VD*ST-ingredient*vitamin dosage*stress time. Differences between ingredients, between vitamin level and stress time, and their interactions are presented following two-way ANOVA.

Table S3. Relative expression of stress-related genes (*sod1, gr, hsp70*) at the end of the confinement stress in fish larvae fed with diets differing in ingredients and vitamin dosage

| **Genes** | ***sod1*** | ***gr*** | ***hsp70*** |
| --- | --- | --- | --- |
| **Ingredients (I)** *p* | *0.990* | 0.641 | ***<0.001*** |
| **FBD** | 1.20±0.68 | 0.64±0.74 | 1.00±0.43 |
| **PBD** | 1.20±0.92 | 0.73±0.84 | 1.36±0.76 |
| **Vitamine dosage (VD)** *p* | *0.175* | *0.461* | ***<0.001*** |
| **1x** | 1.34±1.11 | 0.76±0.90 | 1.42±0.93 |
| **2x** | 1.19±0.71 | 0.71±0.93 | 1.13±0.48 |
| **3x** | 1.06±0.46 | 0.58±0.48 | 1.00±0.30 |
| **Stress time (ST)** *p* | *0.898* | *0.559* | *0.891* |
| **BS** | 1.21±0.64 | 0.73±0.87 | 1.20±0.72 |
| **AS** | 1.19±0.94 | 0.64±0.72 | 1.17±0.56 |
| **Interactions** |  |  |  |
| **I*VD** *p* | *0.290* | *0.102* | ***<0.001*** |
| **I*ST** *p* | *0.179* | *0.708* | *0.135* |
| **VD*ST** *p* | *0.250* | *0.675* | *0.983* |
| **I*VD*ST** *p* | *0.244* | *0.318* | *0.064* |

FBD-fish-based diet, PBD-plant-based diet, BS-before stress, AS-after stress. Data expressed as mean ± SD.

I*VD- ingredient*vitamin dosage, I*ST-ingredient*stress time, VD*ST- vitamin dosage*stress time, I*VD*ST-ingredient*vitamin dosage*stress time. Differences between ingredients, between vitamin level and stress time, and their interactions are presented following two-way ANOVA.
